# Physicians’ Visiting List

**Published:** 1884-12

**Authors:** 


PHYSICIAN'S VISITING LIST. We have received from P. Blakiston, Son & Co., Philadelphia, their Physician's Visiting List for 1885. It contains table of poisons and their antidotes, dose table, list of new remedies, metric system of weights and measures, calendar for 1885–86, Marshall Hall's ready method in asphyxia, Sylvester's method for producing artificial respiration and a diagram of the chest. It is substantially bound and can be easily carried in the pocket. It is a convenient compendium of useful information adapted for every day use, and no physician should be without it.

